# Five‐year trajectories of HbA1c by age, sex, ethnicity and deprivation in adults with newly diagnosed type 2 diabetes: Observational study in England

**DOI:** 10.1111/dom.16288

**Published:** 2025-03-03

**Authors:** Atanu Bhattacharjee, Mohammad R. Ali, David Kloecker, Suping Ling, Gayathri Victoria Balasubramanian, Clare Gillies, Melanie Davies, Kamlesh Khunti, Francesco Zaccardi

**Affiliations:** ^1^ Leicester Real World Evidence Unit, Leicester Diabetes Centre, Leicester General Hospital University of Leicester Leicester UK; ^2^ Population Health and Genomics, Medical School University of Dundee Dundee UK; ^3^ Leicester Diabetes Research Centre, Leicester General Hospital University of Leicester Leicester UK; ^4^ Medical Research Council Epidemiology Unit University of Cambridge Cambridge UK; ^5^ Inequalities in Cancer Outcomes Network Group, Department of Health Services Research and Policy, Faculty of Public Health and Policy London School of Hygiene and Tropical Medicine London UK; ^6^ Joint‐Education Programme Team, Leicester Medical School University of Leicester Leicester UK; ^7^ Chongqing Medical University Chongqing China; ^8^ NIHR Leicester Biomedical Research Centre Leicester UK

**Keywords:** cohort study, database research, population study, primary care, real‐world evidence, type 2 diabetes

## 1 BACKGROUND

The burden of diabetes mellitus (DM) is increasing worldwide, putting significant pressure on healthcare systems. Diabetes is often managed in primary care and includes a significant proportion of adults needing treatment. HbA1c is considered the gold standard for monitoring overall glycaemic level control: while guidelines generally recommend maintaining HbA1c levels below 52 mmol/mol (7%) for most individuals with diabetes, personalized management strategies are advocated.^1^ However, the regular monitoring of HbA1c in real‐world settings differs from that in controlled clinical trials.

This study investigates five‐year HbA1c trajectories in newly diagnosed individuals with type 2 diabetes (T2DM) in routine primary care clinical practice in relation to age, sex, ethnicity and socioeconomic deprivation. We aim to identify disparities in HbA1c trajectories arising from these demographic factors, and to inform the development of more personalized and equitable approaches to diabetes care by identifying subgroups that may require targeted interventions or additional strategies for better diabetes glycaemic management.

## 2 METHODS

We conducted this retrospective observational study, with approval from the Independent Scientific Advisory Committee (ISAC protocol No. 20_122), following the RECORD guidelines (checklist in the Supplementary material).

### 2.1 Data

We used non‐overlapping primary care data available in CPRD GOLD and Aurum. CPRD includes information on sociodemographic, lifestyle, clinical, laboratory, diagnosis and prescription data.^2^, ^3^ We extracted information on adults (≥18 years) with only newly diagnosed T2DM between 1 January 2000 and 5 April 2020 and with at least one measurement of HbA1c during the follow‐up from diabetes diagnosis (from index date) up to 5 years (Figure S1).

### 2.2 Risk factors

Four risk factors, potentially associated with HbA1c trajectories, were considered: age, sex, deprivation and ethnicity. Age was grouped into seven distinct categories based on its distribution: ≥18 to <30, ≥30 to <40, ≥40 to <50, ≥50 to <60, ≥60 to <70, ≥70 to <80 and ≥80 years. Sex was obtained from a binary variable (male or female). We used the index of multiple deprivation (IMD) deciles (least deprived, decile 1; most deprived, decile 10). IMD is a based on a composite score from seven deprivation domains (income, employment, education, health and disability, crime, housing and living environment) from the population of England. While ethnicity was coded as White, Black, South Asian and Others/Unknown using an established algorithm in CPRD.^4^

### 2.3 Statistical analysis

All statistical analyses were conducted in Stata 18. Characteristics at index date are reported as median (interquartile range [IQR]) or number (%) for continuous and categorical data, respectively. Using a linear regression, we estimated associations between HbA1c (outcome) and follow‐up time, accounting for a possible non‐linearity with a spline transformation of time (5 knots located as suggested in Harrell^5^). From these models, we predicted HbA1c over time up to 5 years across levels of age, sex, ethnicity and IMD. Results are reported with 95% robust confidence intervals to account for clusters at the individual level. Additionally, we conducted interaction analyses to evaluate whether HbA1c trajectories differed by age, sex, ethnicity and IMD. Joint significance of these higher‐order interactions (time and a combination of two factors) was tested using a Wald test at a 5% significance level and used robust (clustered) standard errors to account for repeated measures within individuals (Table S1).

## 3 RESULTS

From an initial sample of 788 069 individuals, we excluded 237 421 without linkage to hospital records and 9944 with missing data on ethnicity and deprivation, leaving 540 704 individuals for the analyses reporting 3 886 451 measurements of HbA1c (1 396 464 in GOLD and 2 489 987 in Aurum database) during the 5 years of follow‐up. The mean and median HbA1c were 51.0 (SD 36.4) mmol/mol and 48 (IQR: 41.0–58.5) mmol/mol, respectively. The median age at diagnosis was 62 (IQR: 52–71) (Table 1). There were fewer females (244 760 [45.3%]) than males. The largest proportion of participants (64 732; 12.0%) fell into the second most deprived group (ninth decile), while the smallest proportion (42 611, 7.9%) into the first decile. The majority of the cohort was of White ethnicity (49.9%), followed by Others/Unknown (37.4%), South Asian (8.2%) and Black (4.6%) ethnicity.

**TABLE 1:** Characteristics of the study cohort at type 2 diabetes diagnosis (index date).

	Total (*N* = 540 704)
Age (years)	62.0 (52.0–71.0)
HbA1c (mmol/mol)	48.0 (41.0–58.5)
HbA1c (%)	6.5 (5.9–7.5)
HbA1c (mmol/mol)^a^	51.0 (SD 36.4)
HbA1c (%)^a^	6.8 (SD 3.3)
Age group (years)	
≥18 to <30	6144 (1.1)
≥30 to <40	27 337 (5.1)
≥40 to <50	78 113 (14.4)
≥50 to <60	124 912 (23.1)
≥60 to <70	144 260 (26.7)
≥70 to <80	111 648 (20.7)
≥80	48 290 (8.9)
Sex	
Female	244 760 (45.3)
Male	295 944 (54.7)
Ethnicity	
White	269 572 (49.9)
South Asian	44 201 (8.2)
Black	24 950 (4.6)
Others/Unknown	201 981 (37.4)
Index of Multiple Deprivation (decile)	
1st (least deprived)	42 611 (7.9)
2nd	46 368 (8.6)
3rd	49 805 (9.2)
4th	50 812 (9.4)
5th	50 032 (9.3)
6th	54 720 (10.1)
7th	55 647 (10.3)
8th	62 890 (11.6)
9th	64 732 (12.0)
10th (most deprived)	63 087 (11.7)

*Note*: Characteristics at type 2 diabetes diagnosis. Estimates are reported as median (interquartile range) or number (%).

^a^ Values reported as mean (SD [standard deviation]).

**FIGURE 1:**
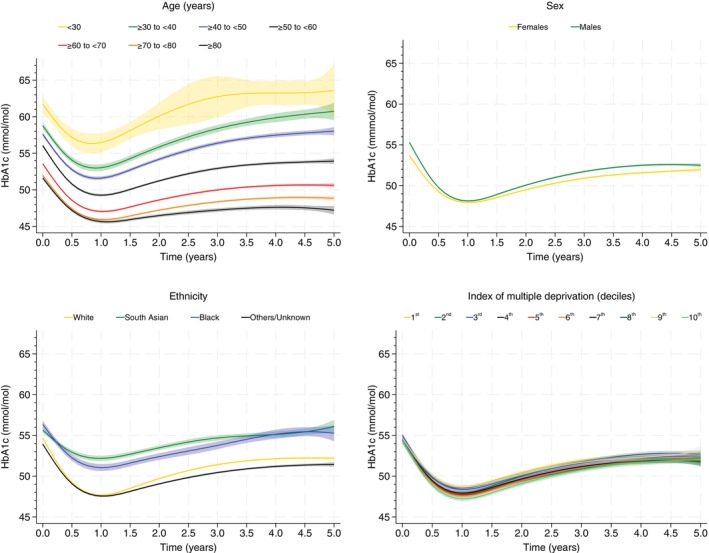
Trajectories of HbA1c across individual characteristics. Estimates are reported as the mean (95% confidence interval, areas). The first decile is the least deprived group, while the 10th the most deprived.

Across all groups, HbA1c levels showed an initial decrease until the first year from the diagnosis and an increase thereafter (Figure 1). The largest differences were observed for age and ethnicity: in terms of age, those between 18 and 30 years had an average HbA1c of 62 (95% CI: 61, 63) mmol/mol (results reported in % in Supplementary text) at diagnosis, 56 (55, 58) at 1 year and 64 (60, 67) at 5 years; corresponding estimates in the oldest group (≥80 years) were 52 (51, 52), 46 (45, 46) and 47 (47, 48). In White and Others/Unknown ethnicities, the trajectories of HbA1c were similar: at diagnosis, 1‐ and 5‐year values were 55 (55, 55), 48 (48, 48) and 52 (52, 52) mmol/mol, respectively, in White; and 54 (54, 54), 48 (47, 48) and 51 (51, 52) in Others/Unknown ethnicity. The levels were similar, albeit higher for Black (56 [56, 57], 51 [51, 51] and 55 [54, 56] mmol/mol at diagnosis, 1 and 5 years, respectively) and South Asian (56 [55, 56], 52 [52, 53] and 56 [55, 57]) ethnicities. Variations were minimal across sex or deprivation levels (Figure 1).

We examined interactions between time and then two demographic factors in separate linear regression models. When combined with time, we found significant interactions with age and sex, age and deprivation, sex and ethnicity (*p* < 0.001) yet found no interactions between age and ethnicity (*p* = 0.130) and sex and deprivation (*p* = 0.180) (see Table S1).

## 4 CONCLUSIONS

While previous studies have summarized HbA1c trajectories in relation to outcomes of glucose‐lowering therapies,^6^, ^7^ we detailed continuous five‐year fluctuations in HbA1c levels across patients' age, sex, deprivation and ethnicity using large, real‐world data. HbA1c decreased from diagnosis of T2DM to 12 months; however, there was a concerning reversal from 1 to 5 years post‐diagnosis seen across all groups. The initial HbA1c improvement (downward trend) may reflect the positive impact of early interventions like medication and lifestyle modifications, and patient adherence. These patterns signify the importance of sustained, long‐term disease management, highlighting the need for regular monitoring of HbA1c levels and possibly compelling modifications in the treatment strategy to maintain optimal blood glucose control over time.

Using the IMD‐2010 index, we found minimal differences in HbA1c levels during follow‐up between the ‘least’ and ‘most’ deprived groups, in contrast to previous findings of clearer associations between deprivation and HbA1c.^8^ When comparing HbA1c by sex, males generally had slightly higher levels of HbA1c than females throughout the follow‐up period, but differences were negligible. Larger differences in HbA1c were observed across ethnic groups: while all groups showed a decrease in HbA1c levels in the first year post‐diagnosis, individuals in the South Asian and Black ethnicity groups showed higher HbA1c from diagnosis compared with the White ethnic group, similar to previous findings.^8^ Further exploration could aid disease management and address any sequelae associated with suboptimal blood glucose management. Finally, our research found that hyperglycaemia at diagnosis and age are inversely associated with HbA1c at diagnosis, highest in the youngest group across and during the 5 years of follow‐up, in line with previous findings.^9^ Particularly concerning was the observation that after 5 years of treatment, the youngest age groups (<40 years) showed higher blood glucose levels than at diagnosis. This finding may be related to the higher rate of complications associated with early‐onset type 2 diabetes (EOT2D).^10^ It also highlights potential limitations in treatment planning, which are mainly based on existing T2D in older populations. These results underscore the need for specific tailored interventions by healthcare professionals for EOT2D.^11^ Further research is needed to clarify the aetiology of the lower HbA1c results over 5 years in the elderly age groups, and if it could be contributing to lower quality of life because of unnecessary polypharmacy.^12^ Moreover, we were surprised to see that there were limited differences in HbA1c across deprivation deciles, contrary to previously published literature.^13^ Further investigation is needed to understand this conflicting finding.

Overall, the comprehensive analysis performed in this study showed differential HbA1c levels in T2DM patients when investigated by age, sex, ethnicity and deprivation; albeit when interaction analyses were conducted, we found significant interactions between some of the different factors and time. One of the main strengths of this study is its use of large real‐world primary care data to track HbA1c levels over a five‐year period. We found that, despite the availability of new treatments over the period of analysis, HbA1c levels remained poor for up to 5 years across all age groups. The findings show how this key clinical biomarker for T2DM management differed by multiple demographic factors. Further research should explore the development of strategies for higher‐risk groups such as EOT2D and Black populations. Targeted interventions and policies to reduce these disparities are needed.

## AUTHOR CONTRIBUTIONS

Francesco Zaccardi, David Kloecker and Suping Ling designed the study. Francesco Zaccardi and Kamlesh Khunti acquired research funding. Francesco Zaccardi defined the clinical codes. Suping Ling extracted the data. Mohammad R. Ali and Atanu Bhattacharjee cleaned the data. Mohammad R. Ali, Francesco Zaccardi and Atanu Bhattacharjee analysed the data. Mohammad R. Ali, Francesco Zaccardi, Atanu Bhattacharjee and Gayathri Victoria Balasubramanian drafted the article. All authors contributed to the interpretation of the data, critically revised the article and approved the final version. Mohammad R. Ali, Francesco Zaccardi and Atanu Bhattacharjee had full access to all the data. Francesco Zaccardi is the guarantor of this work and, as such, had full access to all the data in the study and takes responsibility for the integrity of the data.

## CONFLICT OF INTEREST STATEMENT

Kamlesh Khunti has acted as a consultant, speaker or received grants for investigator‐initiated studies for AstraZeneca, Novartis, Novo Nordisk, Sanofi‐Aventis, Lilly, Merck Sharp & Dohme, Boehringer Ingelheim and Bayer. Melanie Davies has acted as a consultant/advisor and speaker for Eli Lilly, Novo Nordisk and Sanofi, has attended advisory boards for Amgen, AstraZeneca, Biomea Fusion, Carmot/Roche, Sanofi, Zealand Pharma and Regeneron, and as a speaker for AstraZeneca and Boehringer Ingelheim. She has received grants from AstraZeneca, Boehringer Ingelheim and Novo Nordisk. Francesco Zaccardi has received consultancy fees from Daiichi Sankyo, Menarini and Servier for projects not related to this investigation. All other authors declare that there are no relationships or activities that might bias, or be perceived to bias, their work.

### PEER REVIEW

The peer review history for this article is available at https://www.webofscience.com/api/gateway/wos/peer‐review/10.1111/dom.16288.

## Supporting information


**Data S1.** RECORD_checklist HbA1c trajectories v1.0.

**Data S2.** Supplementary text—HbA1c results reported in %.

**Figure S1.** Flow chart of patients from CPRD GOLD and Aurum.

## Data Availability

Data access is through permission from the CPRD only (Protocol No. 20_122); enquiries should be addressed to enquiries@cprd.com.
