# Supplementary material for: Five‐year trajectories of HbA1c by age, sex, ethnicity and deprivation in adults with newly diagnosed type 2 diabetes: Observational study in England
Source: Diabetes Obes Metab. 2025 Mar 3;27(5):2896–900. doi: 10.1111/dom.16288 (PMC11964984; doi:10.1111/dom.16288)
Supplement: Supplementary file 2 — Data S2. Supplementary text—HbA1c results reported in %. [file DOM-27-2896-s001.docx]

**Supplementary text – HbA1c results reported in %**

Those between 18 and 30 years had an average HbA1c of 7.8 (95% CI: 7.7, 7.9) % at diagnosis, 7.3 (7.2, 7.5) at 1 year, and 8.0 (7.6, 8.3) at 5 years; corresponding estimates in the oldest group (≥ 80 years) were 6.9 (6.8, 6.9), 6.4 (6.3, 6.4), and 6.5 (6.5, 6.5).

At diagnosis, 1-year and 5-year HbA1c values were 7.2 (7.2, 7.2), 6.5 (6.5, 6.5), and 6.9 (6.9, 6.9) mmol/mol, respectively, in White; and 7.1 (7.1, 7.1), 6.5 (6.5, 6.5), and 6.8 (6.8, 6.9) in Others/Unknown ethnicity. Levels were similar albeit higher for Black (7.3 [7.3, 7.4], 6.8 [6.8, 6.8], and 7.2 [7.1, 7.3] % at diagnosis, 1 year, and 5 years, respectively) and South Asian (7.3 [7.2, 7.3], 6.9 [6.9, 7.0], and 7.3 [7.2, 7.4]) ethnicities.

**Supplementary Table - Summary of regression results for 2-factor models with time splines**

| Model | Interaction (P value) |
| --- | --- |
| Age + Sex + Time | < 0.001 |
| Age + Ethnicity + Time | 0.130 |
| Age + Deprivation + time | < 0.007 |
| Sex + Ethnicity + Time | < 0.002 |
| Sex + Deprivation + Time | 0.180 |
