# Supplementary material for: Five‐year trajectories of HbA1c by age, sex, ethnicity and deprivation in adults with newly diagnosed type 2 diabetes: Observational study in England
Source: Diabetes Obes Metab. 2025 Mar 3;27(5):2896–900. doi: 10.1111/dom.16288 (PMC11964984; doi:10.1111/dom.16288)
Supplement: Supplementary file 3 — Figure S1. Flow chart of patients from CPRD GOLD and Aurum. [file DOM-27-2896-s003.docx]

# **Figure S1:** Flow chart of patients from CPRD GOLD and Aurum

**AURUM**

**(n = 451,483)**

**GOLD**

**(n = 336,586)**

**- Index date from 1 Jan 2000 to 5 April 2020**

**- ≥ 18 years old at index date***

**- With at least one HbA1c up to 5 years**

**Individuals excluded for missing data on deprivation and ethnicity (n = 9,944)**

**n = 788,069**

**Individuals excluded due to not being linked with HES data (n = 237,421)**

**n = 550,648**

**n = 540,704**

*Index date is the date of type 2 diabetes diagnosis.
